# Structural performance evaluation of electric vehicle chassis under static and dynamic loads

**DOI:** 10.1038/s41598-025-86924-w

**Published:** 2025-02-12

**Authors:** Omar Zamzam, Aly A. Ramzy, Mohamed Abdelaziz, Tamer Elnady, Ayman A. Abd El-Wahab

**Affiliations:** 1 Design and Production Engineering, Faculty of Engineering, Ain Shams University, Cairo, Egypt; 2 Automotive Engineering, Faculty of Engineering, Ain Shams University, Cairo, Egypt

**Keywords:** Structural analysis, Finite element analysis, Electric vehicle, Dynamic analysis, Dynamic load factor, SimSolid, Aerospace engineering, Mechanical engineering

## Abstract

Electric vehicle (EV) production is pivotal in achieving environmental sustainability by reducing greenhouse gas emissions and air pollution. Since the weight of electric vehicles directly influences the energy consumption and driving range of the vehicle, innovative engineers face a significant challenge in designing an optimized vehicle chassis that remains robust under complex loading conditions. This paper focuses on the dynamic analysis of an EV chassis subjected to transient suspension forces due to hitting speed bumps and proposes a load factor between static and dynamic loads. A quarter vehicle model was adopted and solved using MATLAB Simulink to simulate the transient force transmitted to the chassis under different bump dimensions and vehicle speeds. The load was implemented into three different dynamic analysis studies: Front Loading, Rear Loading, and Torsional Loading. Subsequently, static and dynamic analyses were performed using Finite Element Analysis (FEA) with SimSolid software. The results obtained from the dynamic analysis studies showed that the maximum stress was 288 MPa with a safety factor of 1.12, while the maximum stress in the static analysis was 64 MPa with a safety factor of 5.69. Additionally, a load factor of 4.44 between static and dynamic loads was revealed. Based on these findings, the chassis experiences only elastic deformation and is considered safe for practical use.

## Introduction

The electric vehicle chassis plays a vital role in ensuring the overall performance and safety of the vehicle. It acts as a structural backbone to support all the vehicle components together, including the battery pack, electric motor, and suspension system. The innovative design of an electric vehicle chassis strikes a balance between strength, weight reduction, manufacturing feasibility, and performance requirements to deliver a safe and enjoyable driving experience.

The chassis should maintain structural integrity by being sufficiently robust to withstand static and dynamic loads. Static loads are the constant loads acting on the chassis, typically consisting of the payload of the vehicle and the subassemblies mounted on it. The main sources of dynamic loads that contribute to the deformation of the vehicle chassis include torsional loads, which occur when a wheel encounters a bump on the road and cause an increased suspension load at that specific corner of the chassis. Also, bending loads occur when both front wheels or both rear wheels hit a bump. Additionally, longitudinal and lateral loads arise from inertia effects during braking and cornering^1^.

Finite Element Analysis (FEA) is a computer-aided engineering (CAE) tool used to simulate the behavior of a structure under external loads. It breaks down a complex model into smaller, finite-sized elements that together form a mesh that represents the geometry of the structure being analyzed^2^. Engineers widely use FEA to evaluate the mechanical performance of the chassis against different loading conditions.

### Static analysis

Extensive research has been conducted on the structural analysis of vehicle chassis subjected to static loads by FEA using engineering software, including Inventor, SolidWorks, and ANSYS. This process starts with designing and developing the 3D model of the chassis. Next, the chassis material is selected, and static loads are applied. Then the boundary conditions are applied by fixing the suspension mounting points on the chassis, followed by mesh generation for the chassis. Finally, the analysis results, including the stresses induced on the chassis, are compared to the yield strength of the chassis material, and a static safety factor is obtained^3,4^. Several studies have conducted static analysis to evaluate the torsional stiffness of a chassis, defined as the rigidity of the chassis against torsional loads^1^. Torsional stiffness is essential for ensuring proper handling, stability, and overall performance of a vehicle^5^. It can be measured using FEA by applying torque to the front suspension points of the chassis while constraining the rear suspension points. The torsional stiffness is then calculated by dividing the applied torque by the resulting angular displacement^6,7^. Additionally, experimental testing has been conducted on vehicle chassis using strain gauges to measure deformation during static analysis. The results from experimental testing, when compared to those from FEA, showed great consistency, which highlights the reliability of FEA as a simulation tool^8^.

### Dynamic analysis

Dynamic analysis plays a fundamental role to evaluate the structural performance of the chassis. Modal, harmonic and transient analyses are the main types of dynamic analysis which are typically classified based on the nature of the load and response being analyzed. Researchers have performed modal and harmonic analyses using FEA to identify the natural frequencies of the chassis and determine whether they coincide with the excitation frequencies of the road or engine, which can lead to significant vibrations and resonance^9^. Since natural frequencies are determined based on the mass and stiffness of the structure, the results of the analysis have paved the way for modification and optimization in chassis design to change the natural frequencies of the chassis^10,11^. However, a significant oversight in the existing research is the failure to model the chassis under real-world conditions by neglecting the additional mass imposed on the chassis by seats, passengers, and other accessories. This omission could significantly alter the identification of the actual natural frequencies. Consequently, the simulation outcomes fail to accurately represent real-world scenarios^12^.

Transient analysis address the impact of time-varying loads, which can lead to unexpected failures at stress concentration points in the chassis. Such failures often stem from inadequate design strategies for components subjected to time-dependent forces despite their seemingly brief duration. Impact analysis is closely related to transient analysis, focusing on short-duration, high-intensity loads. It holds a pivotal position in vehicle chassis design literature, as it evaluates the crashworthiness of a vehicle. Crashworthiness refers to the ability of the vehicle to protect occupants during a sudden impact^13^. Several studies have utilized FEA to simulate different crash scenarios, enabling the analysis of the structural integrity and deformation patterns of chassis under impact loads. These studies often focus on how the design and material composition of the chassis can absorb and dissipate energy under crash conditions, thereby minimizing the risk of injury to occupants^14,15^. Table 1 shows the typical analyses conducted to evaluate chassis performance, with the purpose and key outputs of each analysis.

**Table 1:** Summary of Analyses performed on chassis.

Analysis Type	Purpose	Key Outputs
Static	Evaluate response to static loads	Stress, strain, displacement, safety factor, Torsional stiffness, Bending stiffness
Transient	Assess behavior under transient, time-varying loads	Peak stress and time, load paths
Modal	Identify natural frequencies	Natural frequencies, mode shapes
Harmonic	Evaluate response to steady-state oscillations (engine, road roughness)	Displacement, velocity, acceleration responses
Crashworthiness	Simulate impact scenarios for safety	Energy absorption, deformation patterns
Fatigue	Predict long-term durability under cyclic loads	Fatigue life, weak points prone to failure

Transient analysis is often overlooked in chassis design, with limited literature available on its application to address dynamic, time-varying loads. Instead of performing detailed transient analysis, designers rely on static analysis and compensate by increasing the static load by a factor to approximate the effects of dynamic forces^16–18^. In the transient analysis by Rodrigues et al.^19^, the performance of the chassis was evaluated under short-duration impact load using a sinusoidal displacement–time function to act like a road bump. However, the source of this function was not specified in the study. Although no stress results were reported, the small displacements observed confirmed the safety of the chassis under dynamic conditions. Ren et al.^20^ emphasized the significance of transient analysis in their study on the design of dump truck chassis. However, they did not specify the source of the applied load or provide details regarding the excitation forces, leaving a gap in understanding the external factors influencing the chassis dynamic performance.

Mohammed et al.^21^ performed a transient analysis using ANSYS software to investigate the potential for failure due to time-dependent inertial forces. The analysis used acceleration data derived from a velocity versus time graph, which represented the vehicle reaching its maximum velocity. The results, presented as a graph of time versus total deformation, showed a maximum deformation of 0.08 mm confirming the structural safety of the chassis. However, the study did not specify the source of the excitation forces, the values of the inertial forces, or any stress results. These limitations leave gaps in understanding the actual stresses experienced by the chassis under transient dynamic loading.

Kishan et al.^22^ used FEA to perform static and transient analyses to evaluate the structural performance of an electric bus chassis frame with different frame sections. In the static analysis, the loads applied were the gross vehicle weight and payload. For the transient analysis, a force–time function was used as the applied load; however, the source of this load was not specified. For a chassis frame constructed with Steel A709M box sections, the stress results from the static analysis were 42.6 MPa, whereas those from the transient analysis reached 214 MPa. Comparing these results to the yield strength of 260 MPa, the static safety factor was calculated to be 6.1, while the dynamic safety factor was 1.2, assuring the safety of the chassis.

### Finite element analysis

A major problem that may arise during simulation using FEA is singularities. They are unrealistic stress concentrations that occur at specific points within the finite element mesh, where the simulation stress results tend to approach infinity^23^. Singularities typically occur due to geometric sharp corners and inadequate mesh resolution^24^. Although there are strategies to address singularities, such as simplifying the model and refining the mesh around critical regions, these approaches can consume a significant amount of time when simulating complex structures and necessitate the expertise of FEA specialists. SimSolid is CAE software developed by Altair Engineering. It is a mesh-free simulation tool that specializes in performing analysis and optimization for various engineering applications including static, modal, and dynamic analysis. The technology used in this software eliminates the need for geometry simplification and meshing, which are the most time-consuming and error-prone operations typically performed in traditional mesh-based FEA software. The foundation of the SimSolid computational engine lies in the pioneering expansions of the theory of external approximations. The theoretical background of this mesh-free method is presented and documented in their whitepaper^25^. Recently, Renault Nissan Mitsubishi Alliance, automotive manufacturers, used SimSolid to perform structural analysis of vehicle components to ensure that they met safety and performance standards^26^. The accurate results obtained, along with significant time savings, encouraged them to extensively integrate this software in their design process loop. SimSolid is rapidly becoming an efficient structural analysis software that has recently been used by researchers in different fields, including mechanical engineering^27,28^, and bioengineering^29,30^.

The aim of this paper is to perform a comprehensive structural analysis of an electric vehicle chassis using SimSolid software, focusing on both static and transient loading conditions. The sources of loads were clearly identified to ensure accurate representation of real-world conditions. Prestressed modal analysis was conducted to identify the natural frequencies of chassis. For the transient analysis, the excitation force was calculated using a quarter vehicle model in MATLAB Simulink, simulating a vehicle hitting different road bumps at different speeds. The simulation results were evaluated from a design perspective to verify the structural safety of the chassis, and a dynamic load factor was deduced to quantify the relationship between static and dynamic loads.

## Methodology

**Fig. 1:**
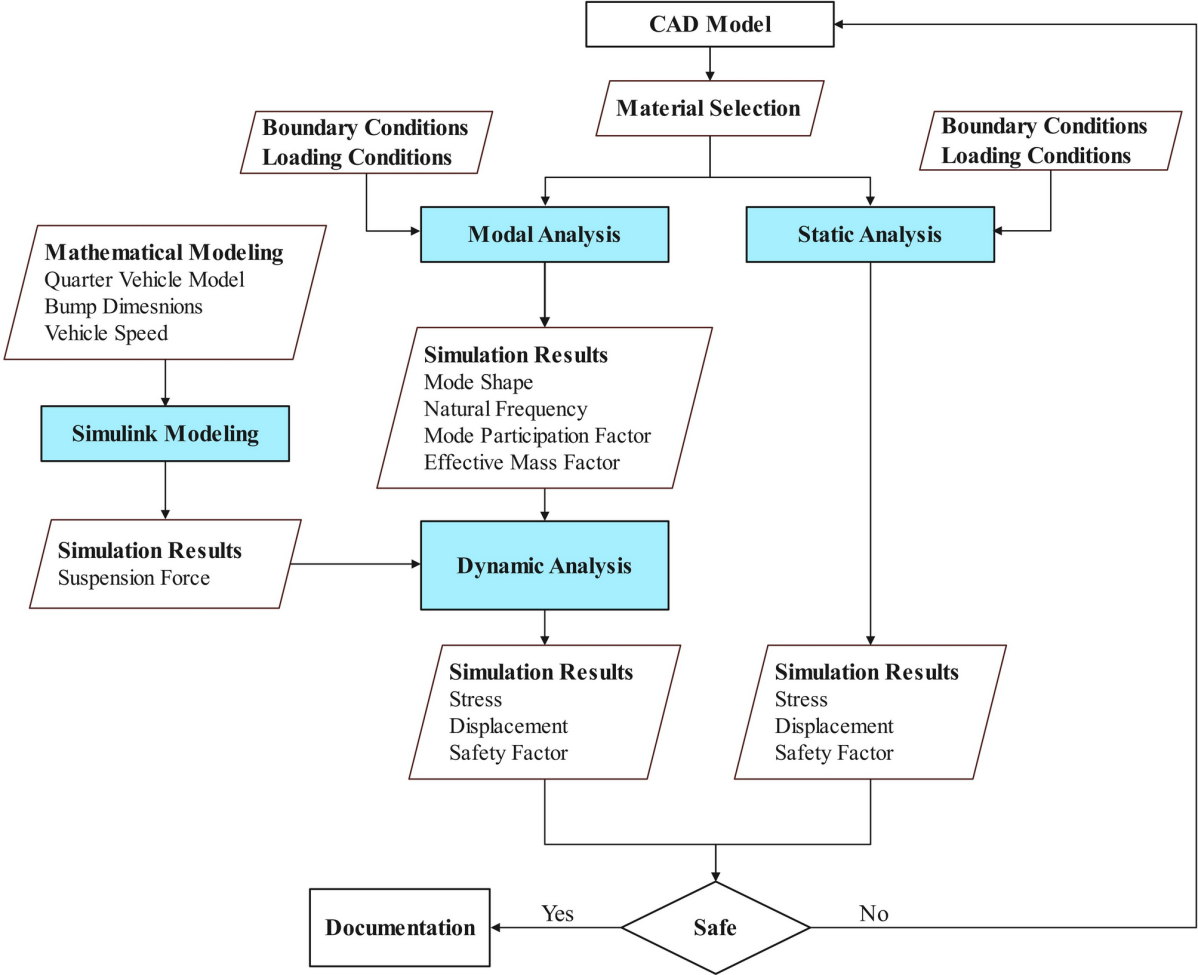
Research process for evaluating the structural integrity of the chassis.

The methodology encompasses static and dynamic analysis using FEA, where dynamic analysis specifically addresses the impact of suspension forces due to speed bumps, which are mathematically modeled and simulated in MATLAB Simulink. Figure 1 illustrates the steps taken throughout the research.

### Vehicle design and materials

**Fig. 2:**
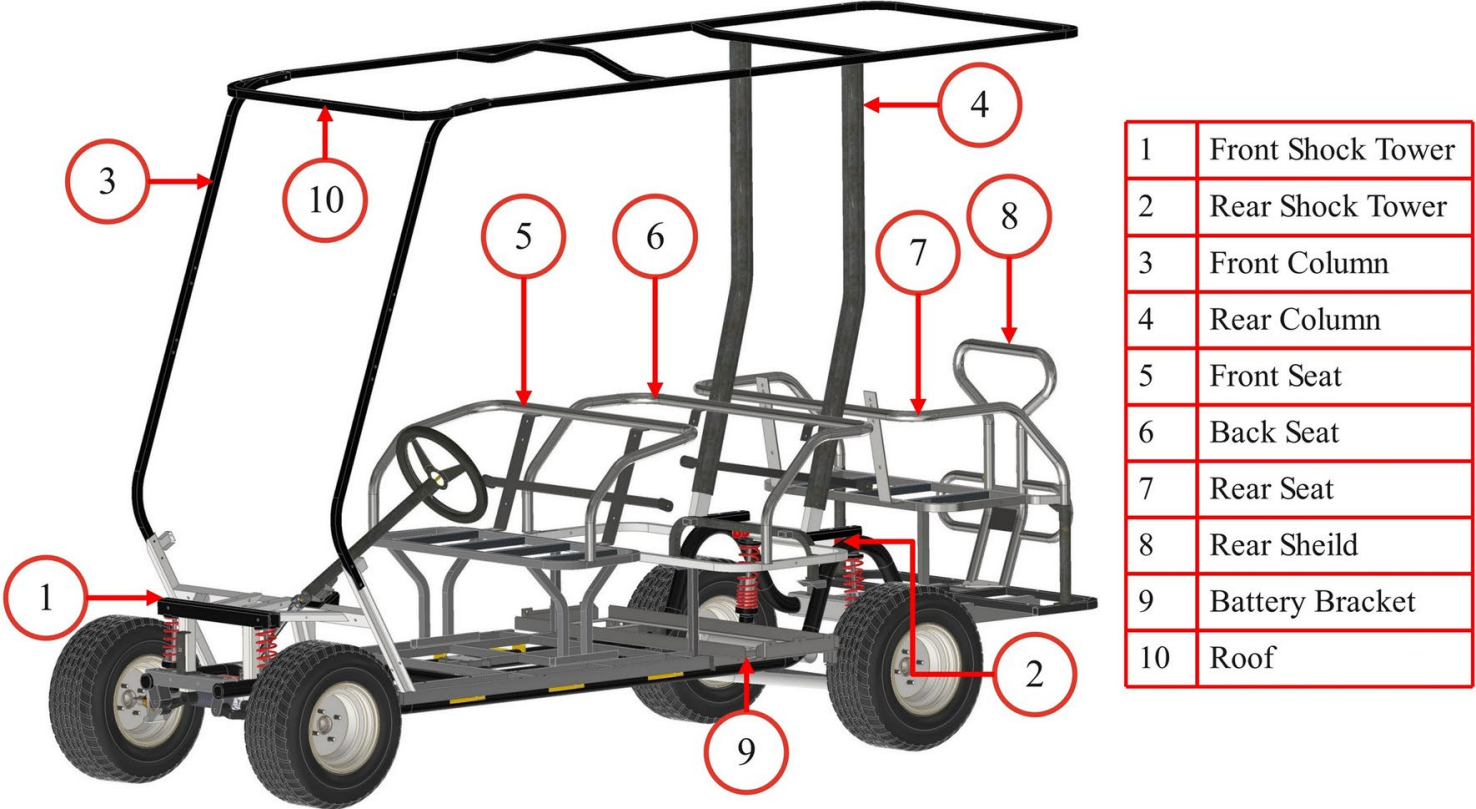
Model of the electric vehicle skeleton with front and rear suspension.

The vehicle mentioned refers to a six passenger electric golf cart to be produced by MATGR company in Egypt^31^. The proposed design of the vehicle chassis, resulting from extensive research and development efforts, is modeled using Inventor software, as illustrated in Fig. 2. The chassis consists of subassemblies welded together, primarily composed of tubes and sheet parts.

The material selected for the chassis is AISI 1018 steel, which is chosen based on its mechanical properties, availability within the country, and cost-effectiveness. Additionally, AISI 1018 steel is known for its high strength and stiffness, enabling it to withstand complex loading conditions experienced by a vehicle chassis^32^. Table 2 presents the material properties of AISI 1018 steel.

**Table 2:** Material properties of AISI 1018 steel^33^.

Parameter	Value
Yield Strength	370 MPa
Tensile Strength	440 MPa
Poisson’s ratio	0.3
Density	2.7 g/cm^3^
Modulus of Elasticity	210 GPa
Shear Modulus	78 GPa

## Dynamic analysis

### Mathematical modeling

**Fig. 3:**
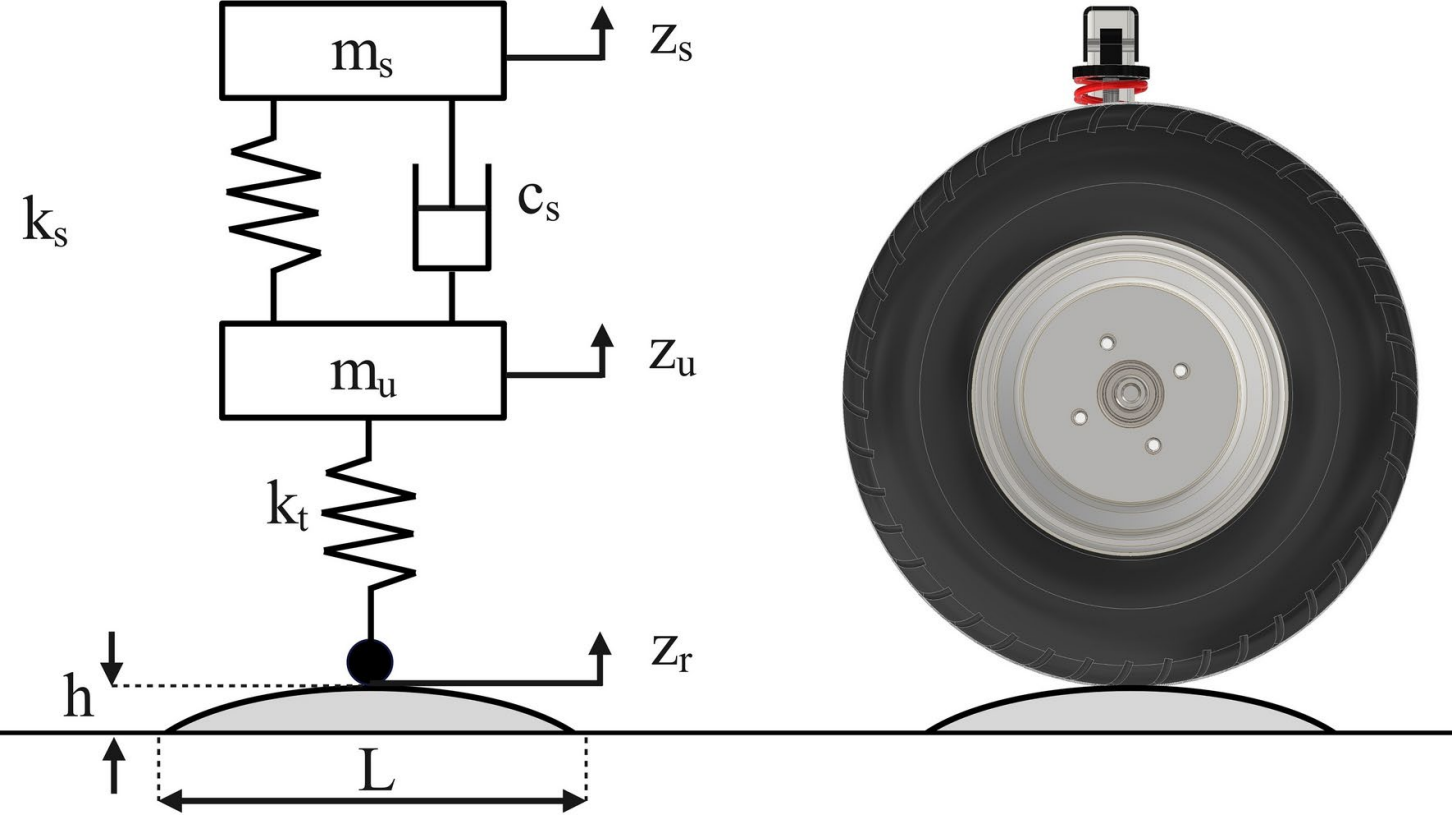
Quarter vehicle model.

A quarter vehicle model is used to study the vertical dynamic forces exerted on the chassis when hitting a speed bump. This model captures the essential characteristics of the suspension system while maintaining computational simplicity^34^. It has been successfully implemented in several studies simulating a vehicle hitting a bump^35,36^ and has demonstrated greater accuracy than the half vehicle model when compared to the full vehicle model^37^. The model neglects lateral dynamics and assumes constant contact between the tyre and the road with no separation. As shown in Fig. 3, the model has two degrees of freedom, where the sprung mass m_s_ represents a quarter of the mass of the chassis, and the unsprung mass m_u_ represents the mass of the wheel assembly. The stiffness of the suspension and its damping coefficient are denoted as k_s_ and c_s_, respectively. Additionally, the vertical stiffness of the tyre is represented as k_t_, while the tyre damping is neglected since it is significantly lower than suspension damping^38^. The vertical dynamic displacements of the model are z_s_ and z_u_, while z_r_ represents the excitation of the system due to variations in the road profile. The spring stiffness and damping coefficient values of the shock absorber adhere to the coilover specifications provided by the manufacturer.

It should be noted that shock absorbers have two damping coefficients: compression damping and rebound damping. Compression denotes the phase where the shock absorber compresses in response to hitting a bump. Rebound is the phase where the shock absorber extends after being compressed to prevent the suspension from bouncing excessively and helps maintain tire contact with the road^39^. The compression damping coefficient is selected in the model, because the force transmitted to the chassis is always greater during compression phase than rebound phase. Table 3 shows the quarter vehicle model parameters according to the designed suspension.

**Table 3:** Quarter vehicle model parameters.

Parameter	Value
Sprung mass (m_s_)	205 kg
Unsprung mass (m_u_)	22 kg
Coilover Spring stiffness (k_s_)	20,600 N/m
Coilover compression damping coefficient (c_s_)	866 N s/m
Tyre Vertical stiffness (k_t_)	200,000 N/m

By applying Newton’s second law of motion, the dynamics of the quarter vehicle model can be described by the following equations^40^:

For the sprung mass:

1 $$\Sigma F=ma$$

2 $${m}_{s}\left({\ddot{z}}_{s}-g\right)={f}_{s}-{f}_{g}$$

where $g$ = gravitational constant, ${f}_{s}$ = suspension force transmitted to the chassis, ${f}_{g}$ = sprung mass gravitational force = ${m}_{s}g$

By eliminating the constants of gravitational acceleration and force, the equation is reduced to:

3 $${m}_{s}{\ddot{z}}_{s}={f}_{s}$$

Hence, the force transmitted to the chassis at the suspension point can be directly calculated through:

4 $${{f}_{s} = k}_{s}({z}_{u}-{z}_{s})+{c}_{S}({\dot{z}}_{u}-{\dot{z}}_{s})$$

By combining Eqs. (3) and (4):

5 $${m}_{s}{\ddot{z}}_{s}={{f}_{s} = k}_{s}({z}_{u}-{z}_{s})+{c}_{S}({\dot{z}}_{u}-{\dot{z}}_{s})$$

For the unsprung mass:

6 $${m}_{u}{\ddot{z}}_{u}{ = {k}_{t}\left({z}_{r}-{z}_{u}\right)-k}_{s}\left({z}_{u}-{z}_{s}\right)-{c}_{S}({\dot{z}}_{u}-{\dot{z}}_{s})$$

**Fig. 4:**
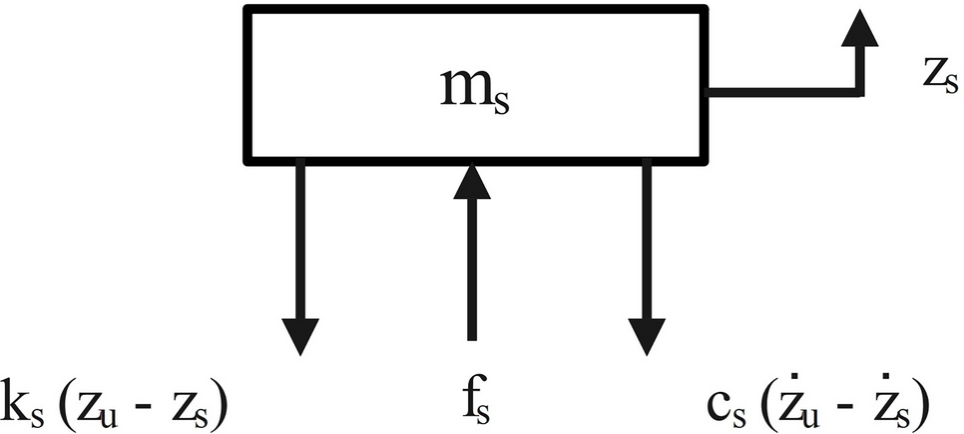
Free-body diagram of the sprung mass.

The free-body diagram shown in Fig. 4 represents the forces acting on the chassis.

**Fig. 5:**
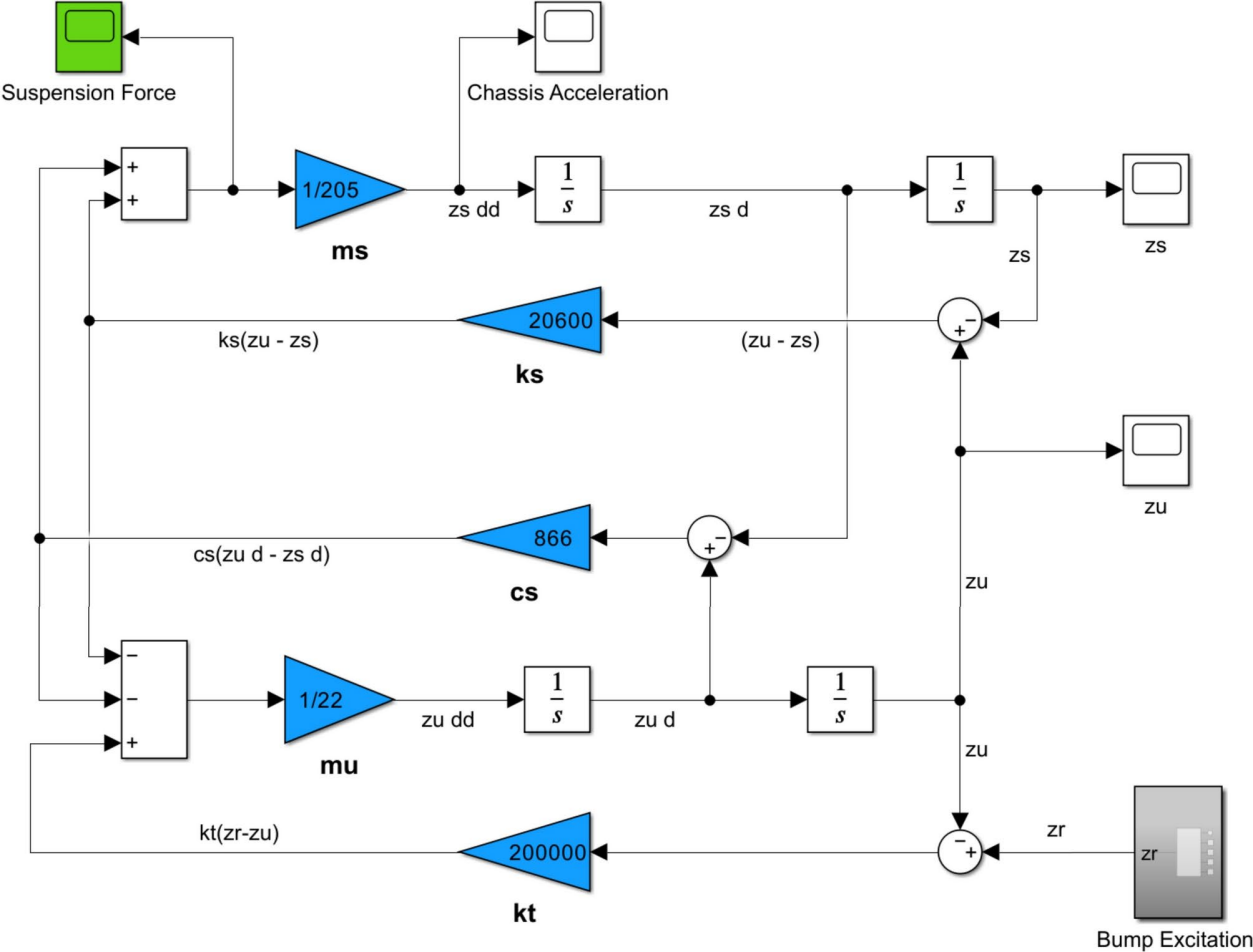
Simulink model block diagram.

Equations (5) and (6) represent the differential equations of motion of the system which are mathematically modeled using MATLAB Simulink software as illustrated in Fig. 5.

The variation in the speed bump profile is modeled by a half sine wave as an input excitation to the system^39,41^. The input excitation is formulated using the following mathematical expressions:

$${z}_{r}(t)=0 for t<\frac{d}{v} and t>\frac{d+L}{v}$$

7 $${z}_{r}\left(t\right)=h\mathit{sin}\left[ \frac{\pi v}{L}\left(t-\frac{d}{v} \right)\right] for t<\frac{d}{v} and t>\frac{d+L}{v}$$

where h and L are the height and length of the bump, respectively. The vehicle velocity is denoted by v. The variable t represents the simulation time starting when the vehicle is about to encounter a bump at a distance d. To simulate the transmitted force under various loading conditions, bump dimensions were selected according to recommended standards^42,43^. Additionally, different vehicle speeds were considered for the simulations, and it should be noted that the chosen values are below the maximum vehicle speed, which is 45 km per hour. Table 4 shows the cases in which the suspension force was solved.

**Table 4:** Cases of different bump specifications and vehicle speeds**.**

Case	Bump Specifications			Vehicle Speed (km/hr)
	Category	Height (mm)	Length (mm)	
1	A	30	400	10
2				20
3				30
4	B	45	500	10
5				20
6				30
7	C	60	600	10
8				20
9				30

**Fig. 6:**
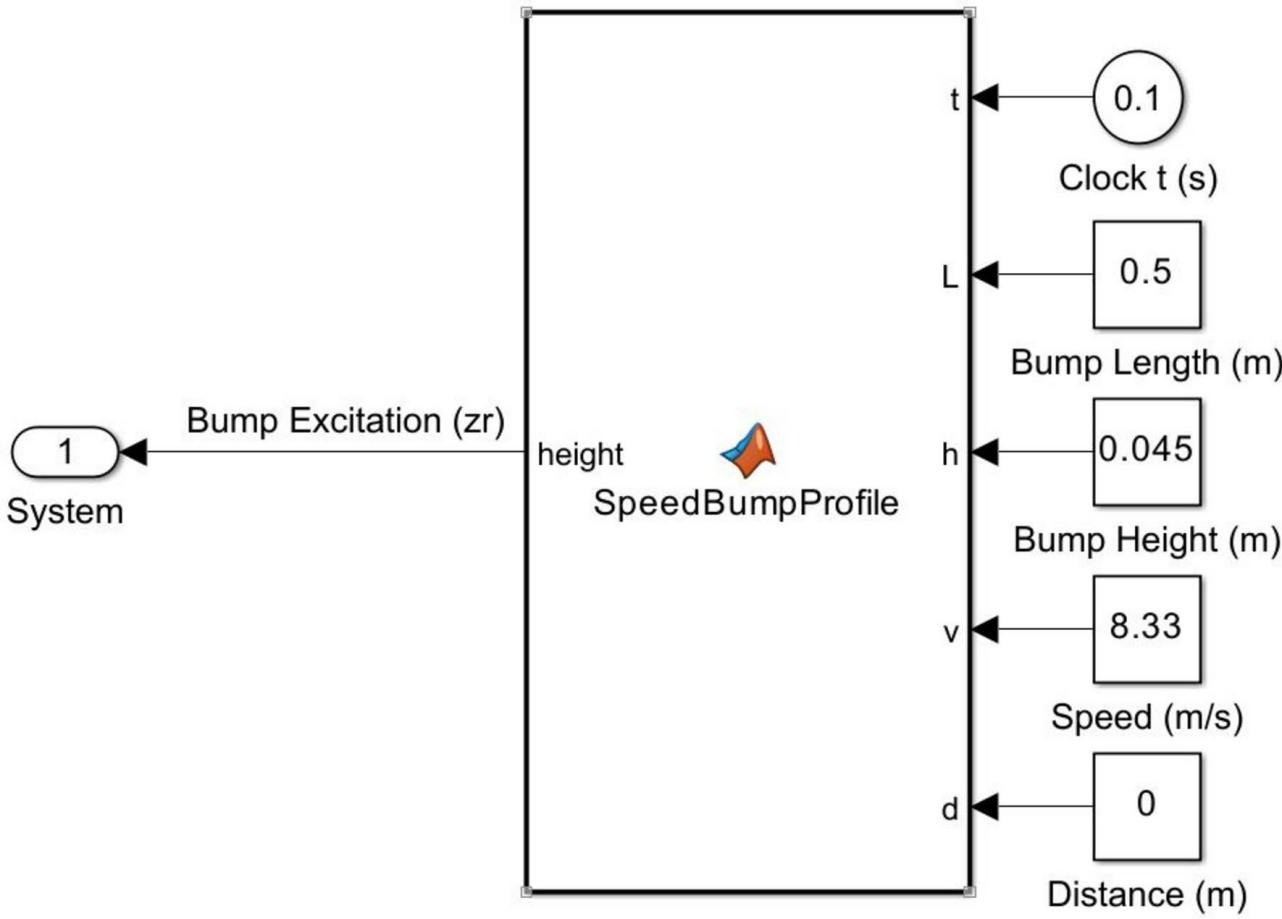
MATLAB function block of bump excitation.

**Fig. 7:**
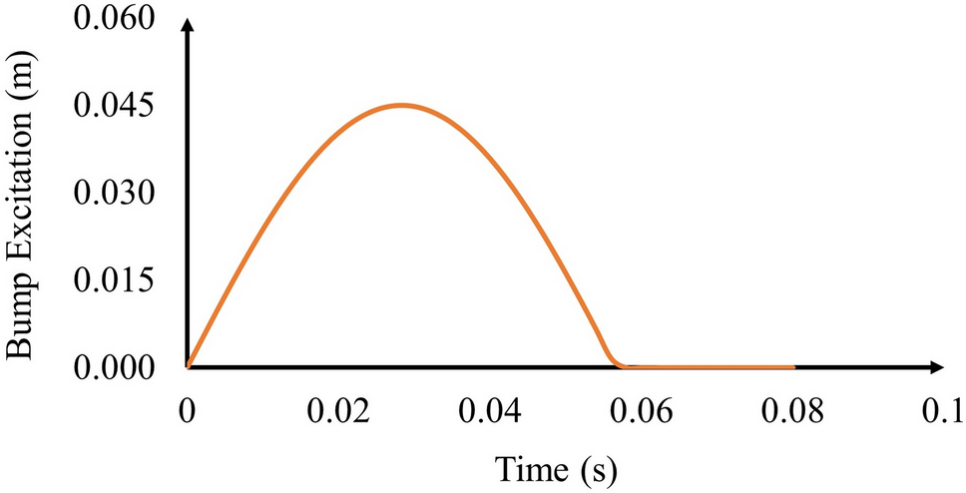
Bump B excitation.

Equation (7) is modeled using the MATLAB function block in Simulink as shown in Fig. 6. Figure 7 shows an example of the generated excitation when the vehicle encounters bump (B) at a speed of 30 km per hour.

The suspension force f_s_ transmitted to the chassis is considered a transient dynamic load, which means that it occurs and vanishes over a range of time. To perform transient analysis, it is first necessary to perform modal analysis.

### Modal analysis

Modal analysis is a process for identifying the dynamic characteristics of a complex structure when it undergoes free vibrations without external forces acting on it. Dynamic characteristics include natural frequencies and vibration mode shapes^44^. Mode shapes refer to how the structure behaves at each natural frequency. The results of natural frequencies at different mode shapes are compared with the suspension transient load frequency to study the possibility of load amplification or resonance. Resonance occurs when the frequency of the applied load aligns with one of the natural frequencies of the structure^45^. This resonance could significantly increase the dynamic stresses and deflections, which may lead to the failure of suspension mounting points on the chassis.

**Fig. 8:**
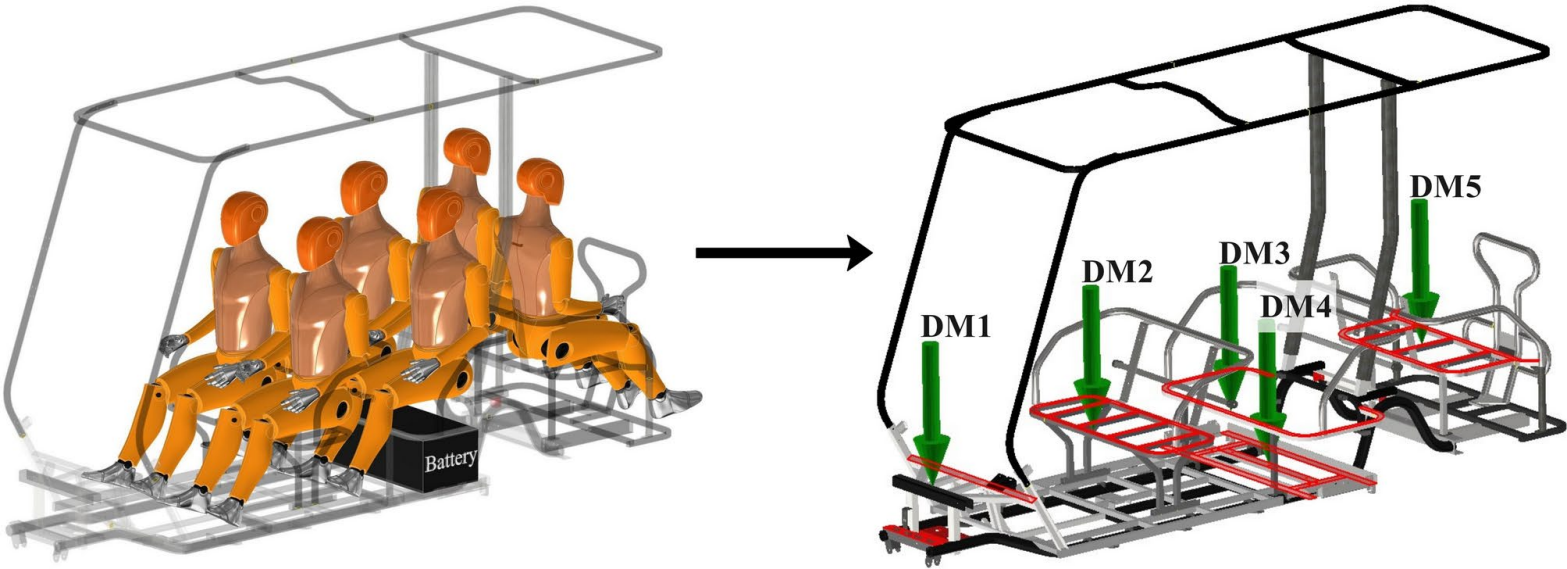
Visualization of the main sources of mass acting on the chassis.

The CAD model was exported to SimSolid software. Modal analysis is performed in free-free settings, where analysis is conducted on the chassis that is unconstrained at both ends, allowing it to move freely in all degrees of freedom. To ensure accurate modal analysis, it is crucial to model the chassis with actual mass and stiffness parameters and distributions. Therefore, the chassis must be preloaded, or in other terms, prestressed^12,46^. This is achieved by applying a series distributed masses on different parts of the chassis, as shown in Fig. 8. These masses substitutes for the effective contributing weights of passengers, the battery, and other subassemblies. It is worth noting that prestressing the structure affects only the natural frequencies of the structure and has no impact on the mode shapes^12,47^. The detailed sources of mass are listed in Table 5.

**Table 5:** Sources of distributed mass.

Distributed Mass	Mass (kg)	Main Source
1	50	Front assembly parts
2	200	Front seat passengers
3	200	Back seat passengers
4	100	Battery
5	200	Rear seat passengers

For a complex structure, the number of vibration modes to be solved is determined based on two factors: the modal participation factor and the effective mass factor^48^. The modal participation factor is a non-dimensional measure of the degree to which a specific mode influences the response of the chassis when it is subjected to excitation in a particular direction. While the effective mass factor for each mode indicates the amount of mass involved in that mode in a certain excitation direction^49^. This amount is expressed as a percentage of the overall mass of the structure. If a mode accounts for more than 1% to 2% of the total effective mass, it should be examined^50^.

### Transient analysis

The suspension force results, obtained from the Simulink model, were exported to SimSolid software as a force–time function. This function simulates the force transmitted to the chassis during the compression of shock absorbers when the vehicle encounters a bump. This transient force was applied as a vertical load in three separate FEA dynamic studies:

- Front Loading Study: Force applied at each front shock tower to examine the impact on the chassis when the front tires encounter a bump.
- Rear Loading Study: Force applied at each rear shock tower to analyze the impact on the chassis when the rear tires encounter a bump.
- Torsional Loading Study: Force applied at the right front shock tower only to analyze the chassis when only one front tyre encounters a bump.

**Fig. 9:**
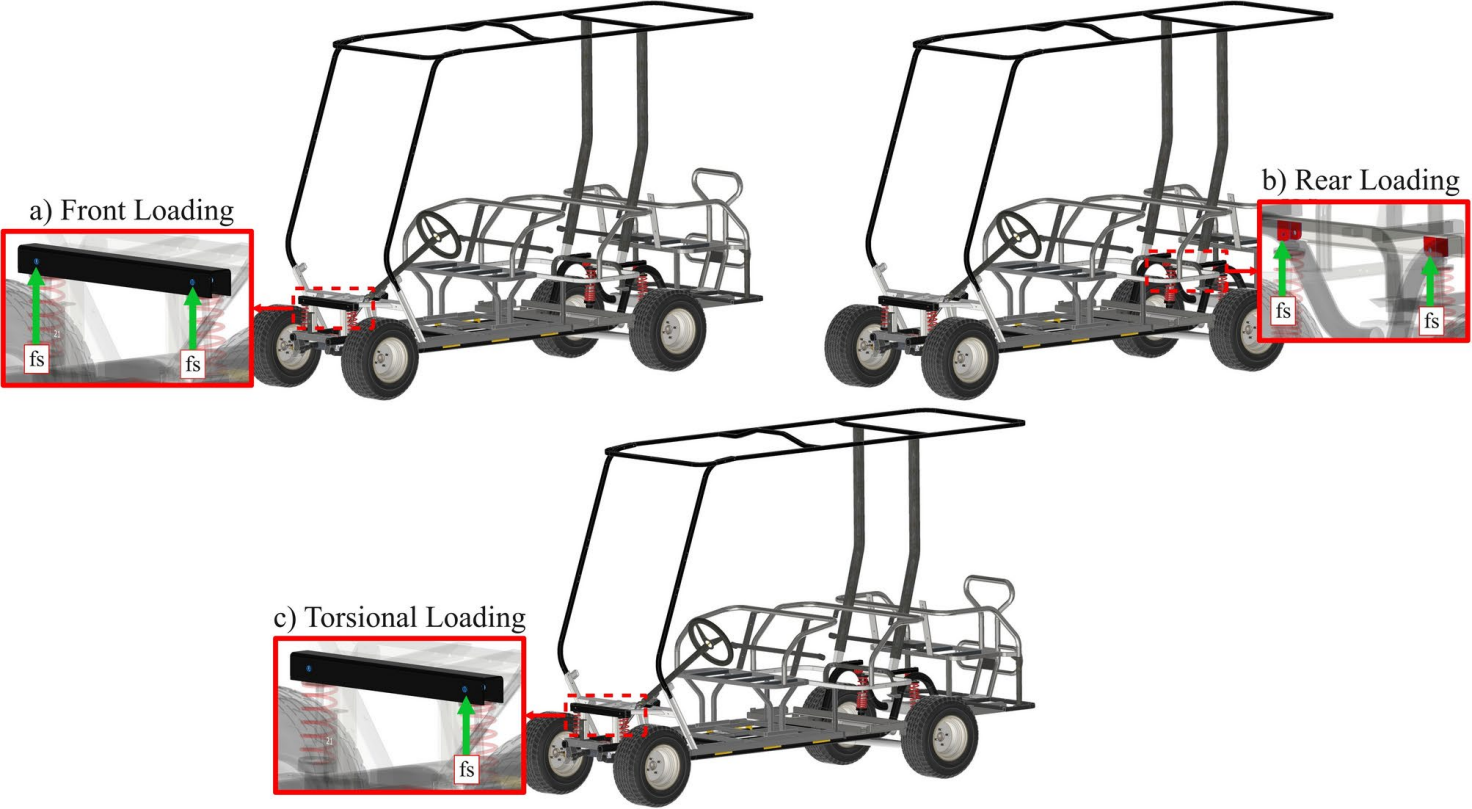
Dynamic analysis: (**a**) Front loading; (**b**) Rear loading; (**c**) Torsional loading.

Figure 9 displays the different dynamic loading cases. The damping ratio of the chassis was set to 0.02, which is a typical value for welded steel structures^51^. It is assumed that the vehicle is traveling at a constant speed and that all tires of the vehicle are in contact with the road in all studies.

#### Static analysis

**Fig. 10:**
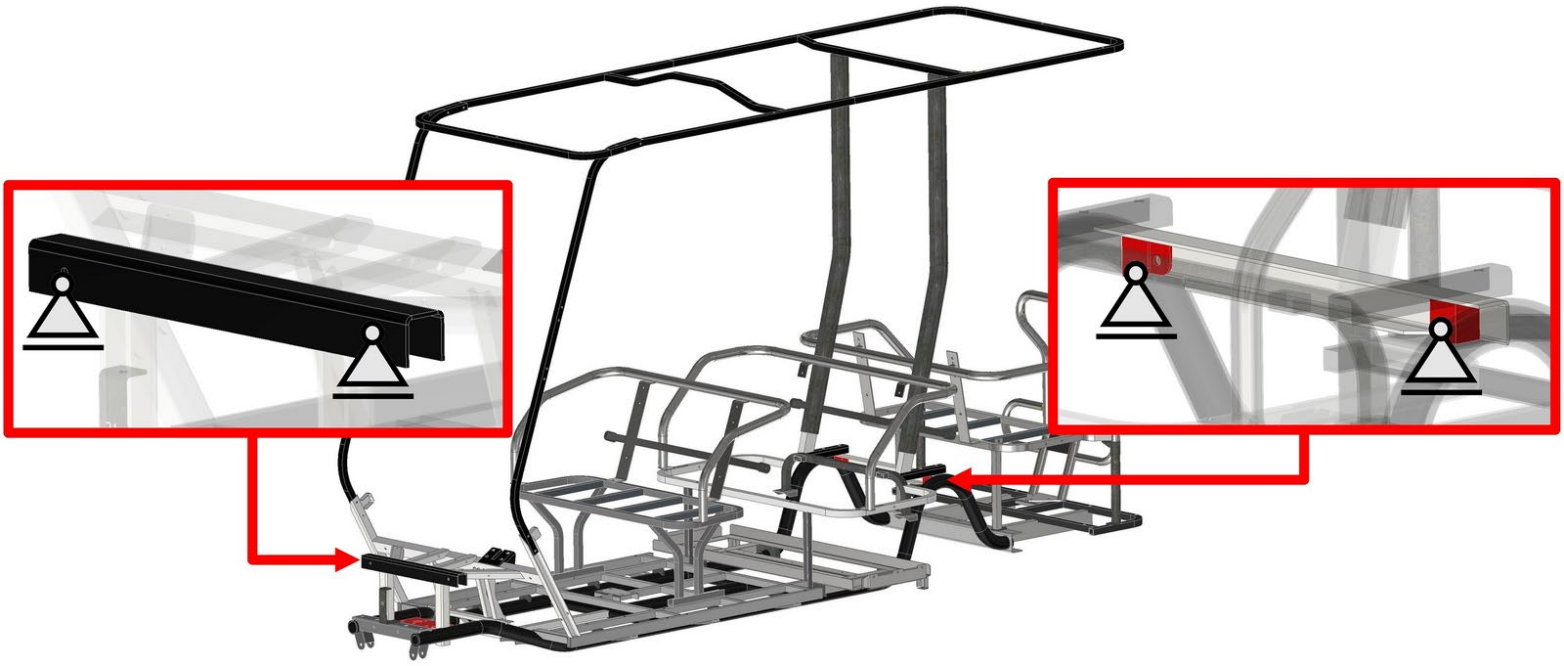
Static analysis boundary conditions.

A static simple bending analysis and simulation were conducted on the chassis of the vehicle using SimSolid software. Static vertical loads were applied to the chassis, assuming the chassis is simply supported at the wheels, following the standard guidelines used in the analysis of vehicle structures^1^. The vertical loads applied were the same as the distributed loads illustrated in Fig. 8. Boundary conditions in the form of fixed constraints are applied to the shock towers where the shock absorbers are connected, as shown in Fig. 10.

## Results and discussion

### Suspension force

**Fig. 11:**
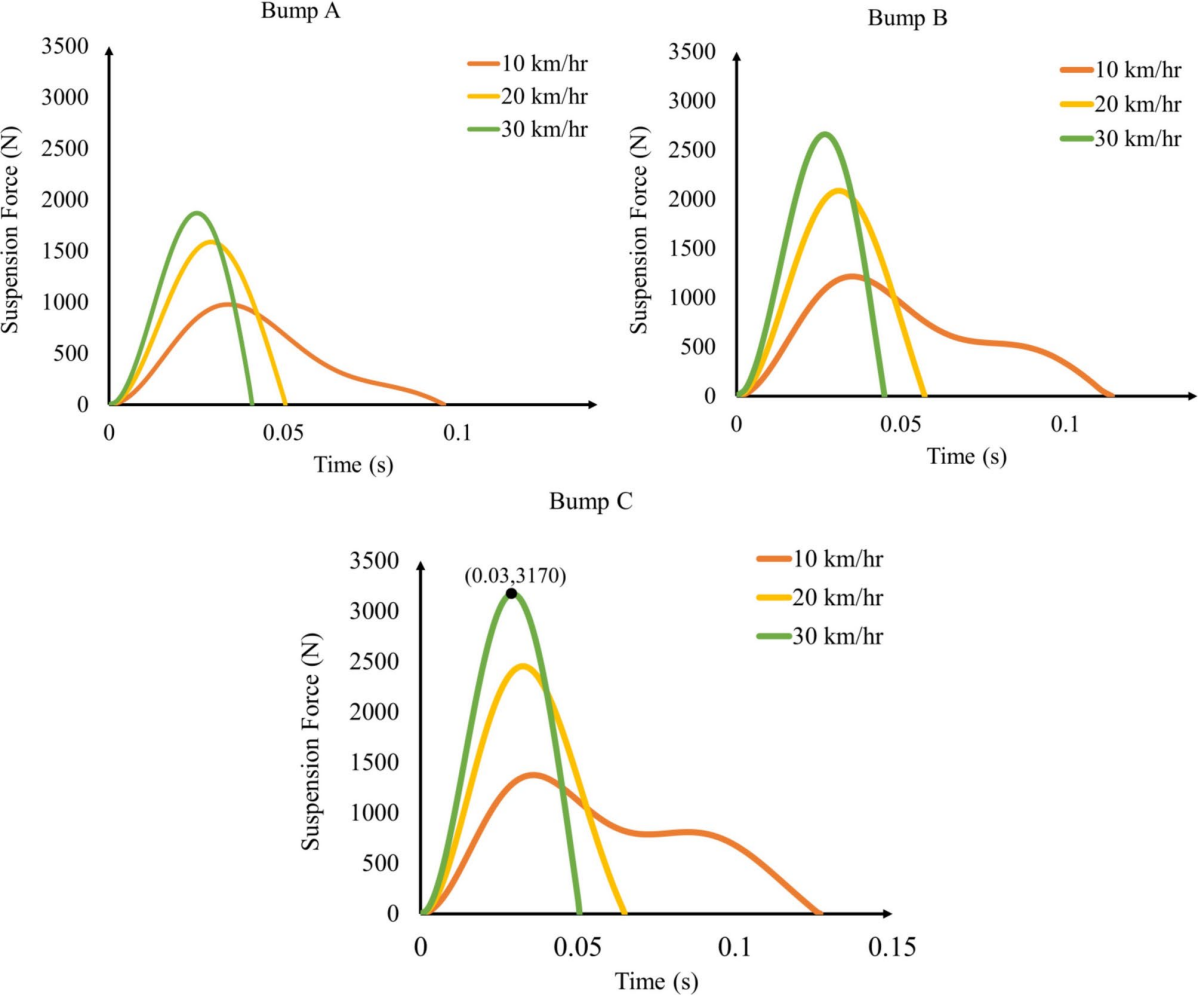
Force–time functions for different bump categories and vehicle speeds.

The solved results of the suspension force for different iterations using the Simulink model are presented in Table 6. Figure 11 shows and compares the suspension force–time function for each case, illustrating their growth and decay over a certain time range. These results reveal how variations in vehicle speed and bump characteristics influence the dynamic loads transmitted to the chassis. A clear trend is observed across all bump categories: as vehicle speed increases, the suspension force also increases. Additionally, it can be observed that vehicles traveling at the highest speed in each bump category experience the highest suspension force within the shortest time.

**Table 6:** Suspension force results for different cases.

Case	Bump Category	Vehicle Speed (km/hr)	Suspension force (N)
1	A	10	980
2		20	1590
3		30	1870
4	B	10	1210
5		20	2080
6		30	2600
7	C	10	1380
8		20	2450
9		30	3170

The maximum suspension force among all the cases occurred in case 9, with a maximum force of 3170 N. This was expected, as this case involved harsh conditions with the largest bump dimensions, measuring 60 mm in height and 600 mm in length, and a vehicle speed of 30 km per hour. This maximum force peaked at nearly 0.03 s and vanished within a total of 0.05 s. Which indicates the importance of designing the chassis to accommodate such short-time but intense loading.

On the other hand, the lowest suspension force occurred in Case 1, when the vehicle encountered Bump A (with a height of 30 mm and length of 400 mm) at a speed of 10 km per hour, resulting in a force of 980 N. This outcome aligns with expectations, where lower suspension force transmitted to the chassis occurs at smaller bump dimensions and lower vehicle speeds. The suspension force–time function results will be used in the dynamic analysis to study their effect on the stresses induced in the chassis, which directly assess the structural integrity of the chassis.

### Modal analysis

The first six modes are rigid body modes with natural frequencies equal to zero. These modes represent pure translation and rotation motion, and in an idealized free-free condition, there is no stiffness associated with these rigid body motions^11^. These modes are neglected, and the only fourteen subsequent vibration modes are studied since they contain the modes with the highest mode participation factor and effective mass factor. Table 7 presents the results of the modal analysis. It shows the natural frequency for each vibration mode, along with the corresponding mode participation factor and mass effective factor for various excitation directions.

**Table 7:** Natural frequencies of the chassis.

Vibration mode	Frequency (Hz)	Mode participation factor			Effective mass factor (%)		
		X	Y	Z	X	Y	Z
7	6.34	1.00	0.00	0.00	99.81	0	0
8	8.29	0.00	0.00	0.00	0	0	0
9	9.52	0.04	0.00	0.00	0.19	0	0
10	10.97	0.00	0.00	0.00	0	0	0
11	11.71	0.00	0.00	0.00	0	0	0
12	12.40	0.00	0.00	0.00	0	0	0
13	13.72	0.00	0.04	0.00	0	0.19	0
14	14.84	0.00	0.05	0.00	0	0.26	0
15	15.56	0.00	1.00	0.00	0	99.55	0
16	16.33	0.00	0.00	0.00	0	0	0
17	16.90	0.00	0.00	0.00	0	0	0
18	17.88	0.00	0.00	0.00	0	0	0
19	18.81	0.00	0.00	0.00	0	0	0
20	20.29	0.00	0.00	1.00	0	0	99.73

**Fig. 12:**
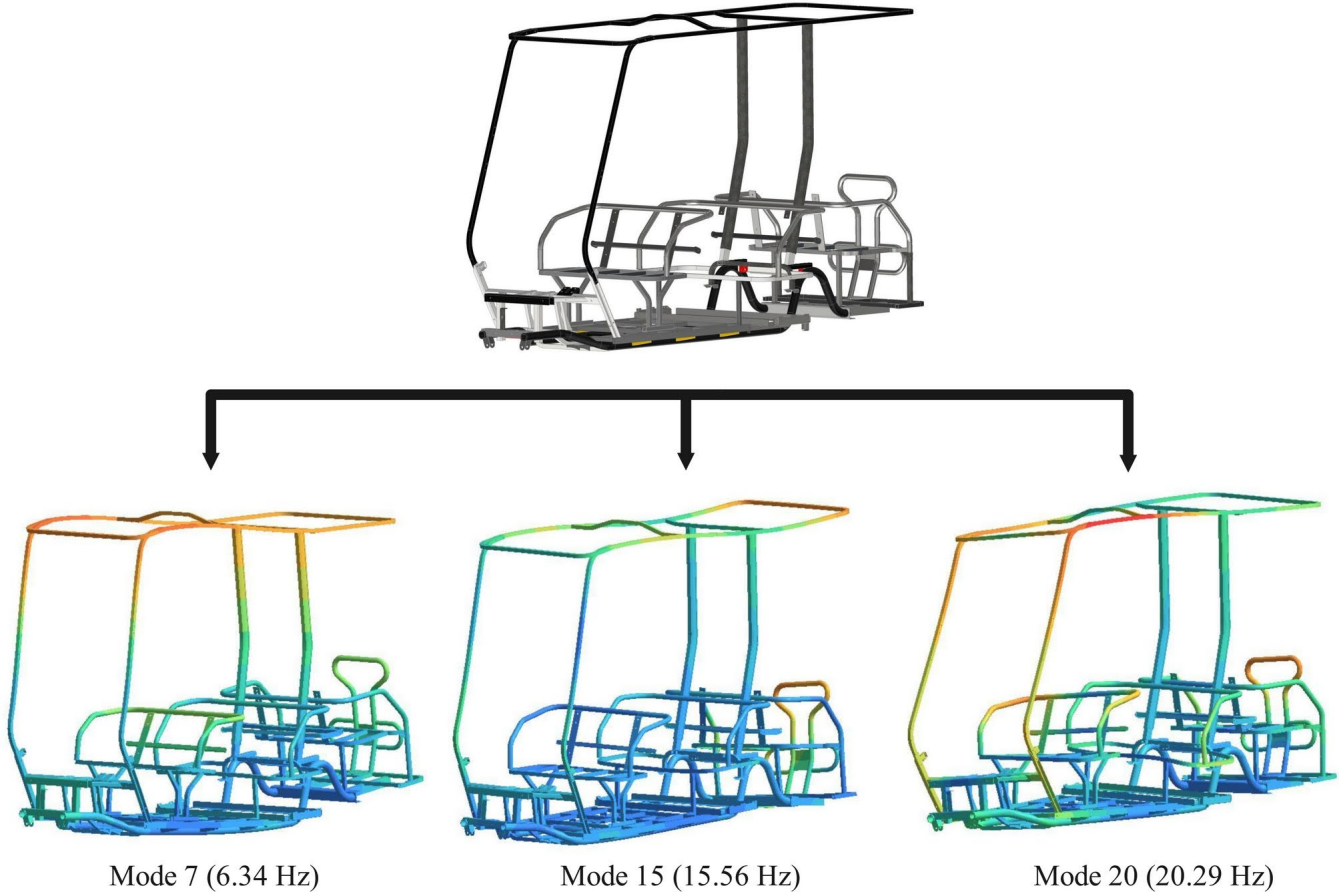
Critical mode shapes deformation.

The critical vibration modes are 7, 15, and 20, as they have the highest mode participation factor and effective mass factor in the x, y, and z directions, respectively. Their natural frequencies range from 6 to 21 Hz. Figure 12 displays the mode shapes for these critical vibration modes, where modes 7 and 15 exhibit deformation due to bending, while mode 20 experiences torsional deformation. Load amplification and resonance could occur if the frequency of the transient load coincides with the natural frequency of these three critical modes.

### Dynamic analysis

The von Mises stress is a measure of the equivalent stress used to evaluate whether a ductile material will plastically deform under complex loading conditions. The von Mises stress is determined based on the principal stresses within the material, including both the maximum and minimum normal stresses^52^. Displacement refers to the deformation experienced by a structure under applied loads. It represents the change in position of the structure’s mesh points (nodes) from their original positions.

It is crucial to calculate the safety factor in both dynamic and static analyses to evaluate the structural integrity of the chassis. However, this process is quite challenging for several reasons. Firstly, the main dynamic load, the suspension force, varies across nine cases. Additionally, this load was applied in different configurations across three dynamic loading studies. Moreover, in dynamic analysis, since the load applied is time-dependent, it results in time-dependent stress on the chassis. This means each component of the chassis experiences a specific peak stress at a certain time due to dynamic load transfer. To simplify the process of calculating the safety factor, the suspension force obtained from the Simulink model that results in the highest von Mises stress and displacement in the Front Loading study will be used in Rear Loading and Torsional Loading studies. Therefore, for each dynamic study, there are calculations of the safety factor based on the peak von Misses stress.

In summary, three dynamic safety factors were calculated for each dynamic loading study and the yield strength of the material, and a single static safety factor based on the yield strength of the material. Finally, a load factor is evaluated as the ratio between the maximum dynamic von Mises stress and the maximum static von Mises stress to propose a ratio between the dynamic and static loads.

### Front loading study

**Fig. 13:**
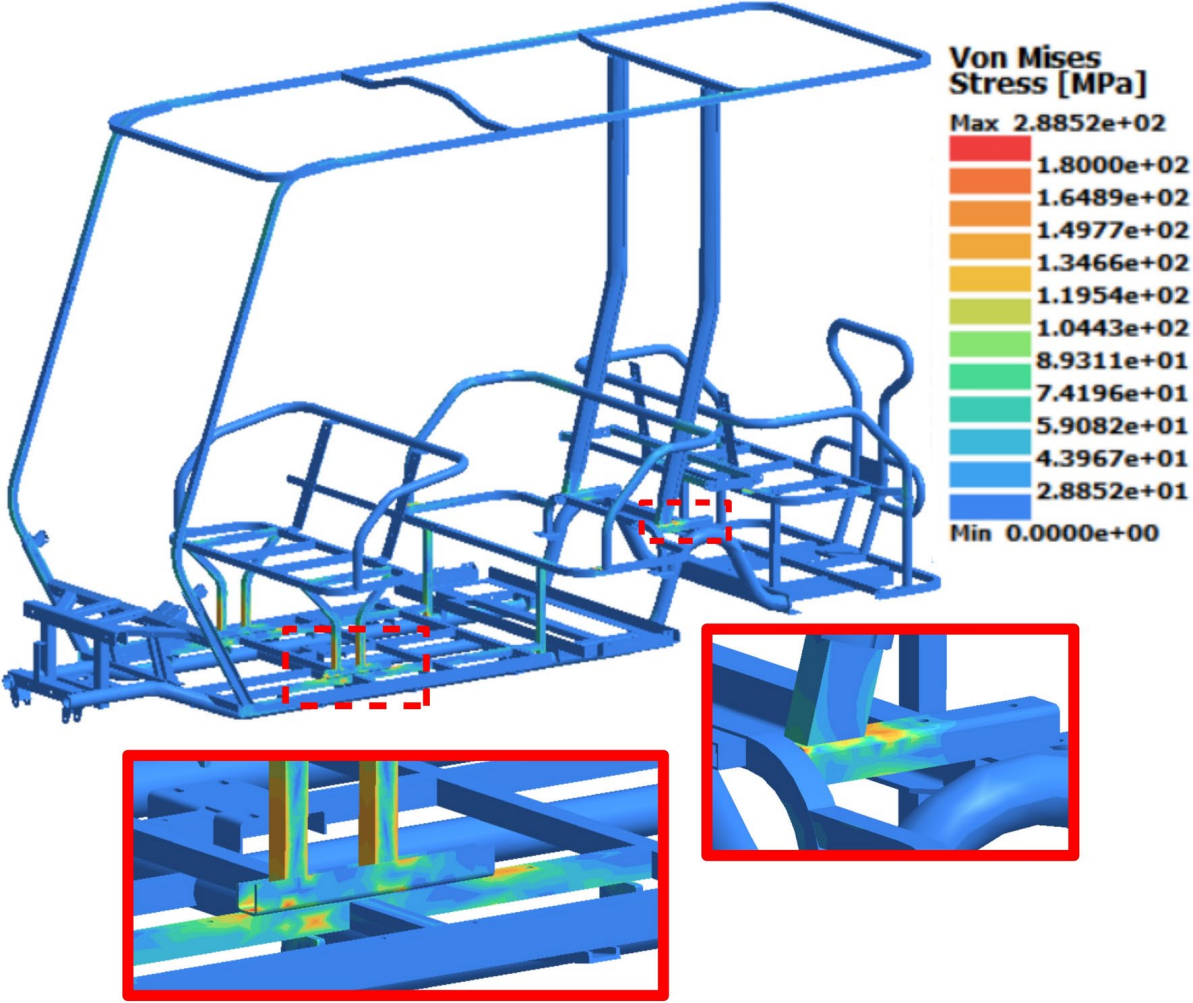
Stress distribution results at 0.13 s for Case 9.

Transient analysis was conducted individually for each case due to variations in their force–time functions. The results of the transient analysis show that case 9 had the highest von-Mises stress of 289 MPa, which occurred at 0.13 s. Although the transmitted force vanished at 0.05 s, the effects of dynamic load transfer and chassis vibration resulted in peak stress after the vehicle hit the bump. Thid maximum von Mises stress values was mainly concentrated on the base of the front seat, as illustrated by the color coding in the legend of Fig. 13. Additionally, stress concentrations reaching 210 MPa were observed on the bracket that connects the back seat to the rear shock towers, while the rest of chassis exhibited minimal stresses (blue colored). By comparing the maximum stress of 289 MPa to the yield strength of the material used (370 MPa), a dynamic safety factor of 1.28 is evaluated.

**Fig. 14:**
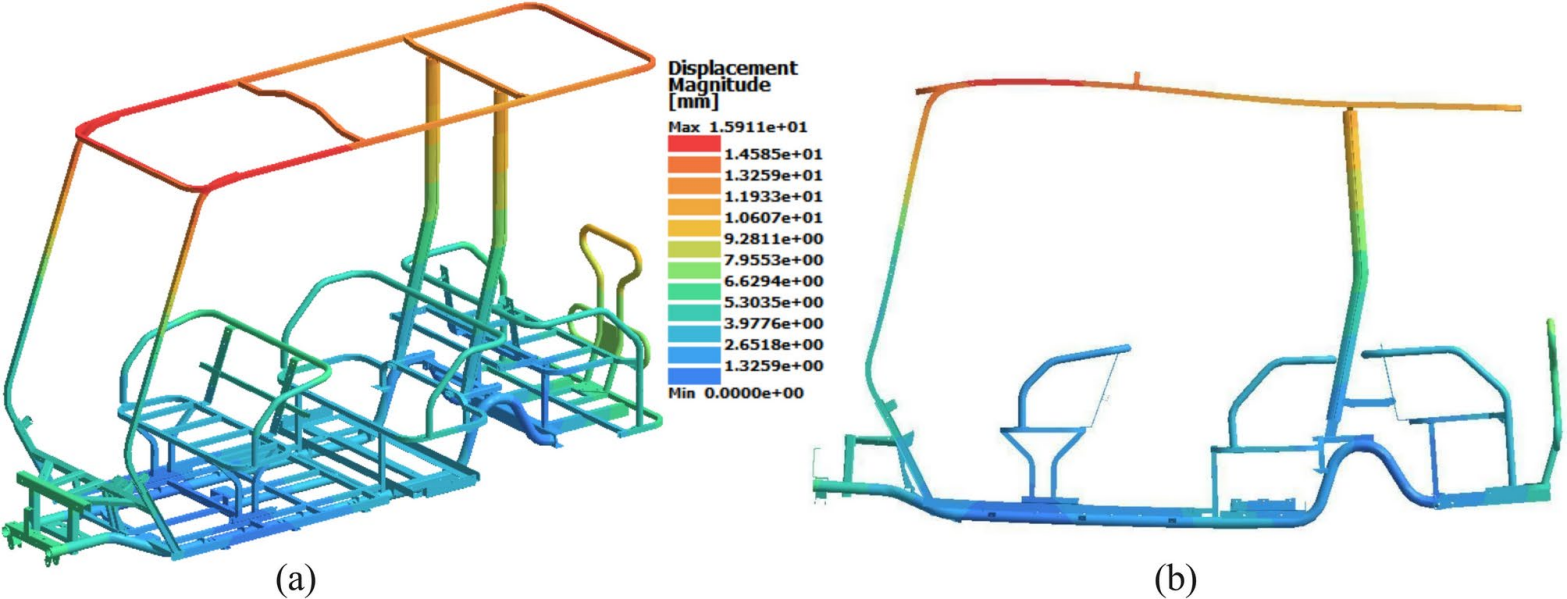
Displacement results for Case 9 in the Front Loading Study. (**a**) Isometric view (deformation scale 1:1), (**b**) Side view (deformation scale 20:1).

The displacement results were determined to simulate the deflection and evaluate the stiffness of the chassis. As expected, Case 9 also had the maximum displacement, with a value of 15.9 mm at 0.07 s. The front part of the roof exhibited the maximum displacement, as illustrated in Fig. 14. Enhancing roof rigidity against applied loads could be achieved by stiffening the roof structure, such as by optimizing tube dimensions or by welding an additional cross member.

The peak results of the von Mises stress and displacement for all the cases are shown in Table 8. There is a clear trend as the suspension force increases, the stress and displacement results increase. Notably, when the chassis was subjected to the lowest suspension force in case 1 resulted in the lowest stress and displacement results.

**Table 8:** Front loading study results.

Case	Suspension force (N)	Von misses stress (MPa)	Maximum displacement (mm)
1	980	89	6.5
2	1590	144	7.9
3	1870	154	8.1
4	1210	108	8.6
5	2080	195	11.5
6	2600	230	11.9
7	1380	126	10.6
8	2450	230	14.1
9	3170	288	15.9

Since Case 9 exhibited the highest von Mises stress and displacement among all examined cases when subjected to the highest suspension force obtained from the Simulink model, this force value will be used in subsequent studies: Rear Loading and Torsional Loading.

### Rear loading study

**Fig. 15:**
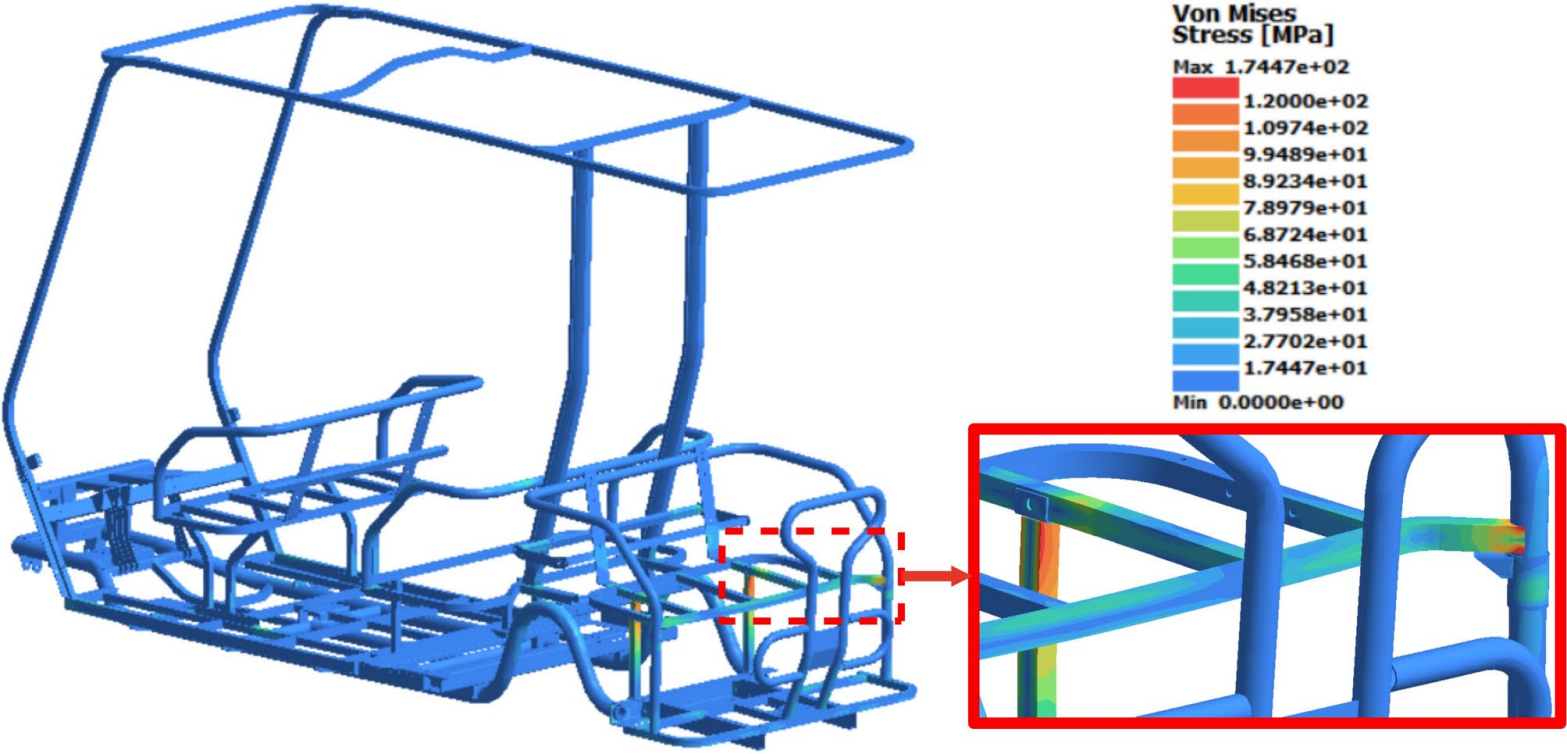
Stress distribution results of the rear loading study.

**Fig. 16:**
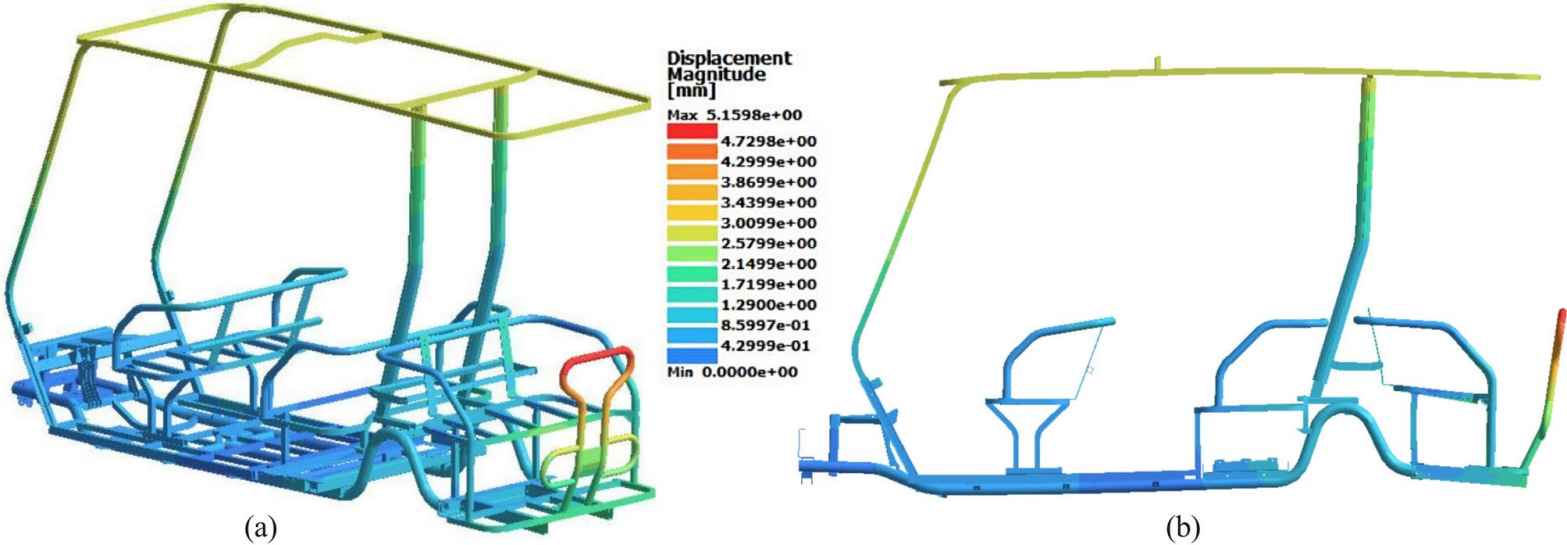
Displacement results of the rear loading study. (**a**) Isometric view (deformation scale 1:1), (**b**) Side view (deformation scale 20:1).

Figure 15 illustrates the stress distribution results on the chassis at peak time. The transient analysis results indicate that the square tubes of the rear seat were subjected to the highest stress, reaching 175 MPa at 0.036 s. A dynamic safety factor of 2.12 is calculated, when the maximum stress result is compared to yield strength of the material used. Moreover, the most significant displacement was observed at the rear shield of the chassis, with a peak value of 5.2 mm occurring at 0.038 s, as shown in Fig. 16.

Notably, in the front loading study, the maximum displacement occurred at the front part of the roof as a result of the vehicle hitting a bump with the front tires. This highlights the roof sensitivity to dynamic loads transmitted through the front of the chassis. In the rear loading study, when the vehicle hit the bump with the rear wheels, the maximum displacement was observed at the rear shield, which is mounted at the rear end of the chassis. This behavior indicates that displacement is directly influenced by the location of load application, with the structure deforming most significantly in the least rigid parts of the chassis closest to the point of impact.

### Torsional loading study

**Fig. 17:**
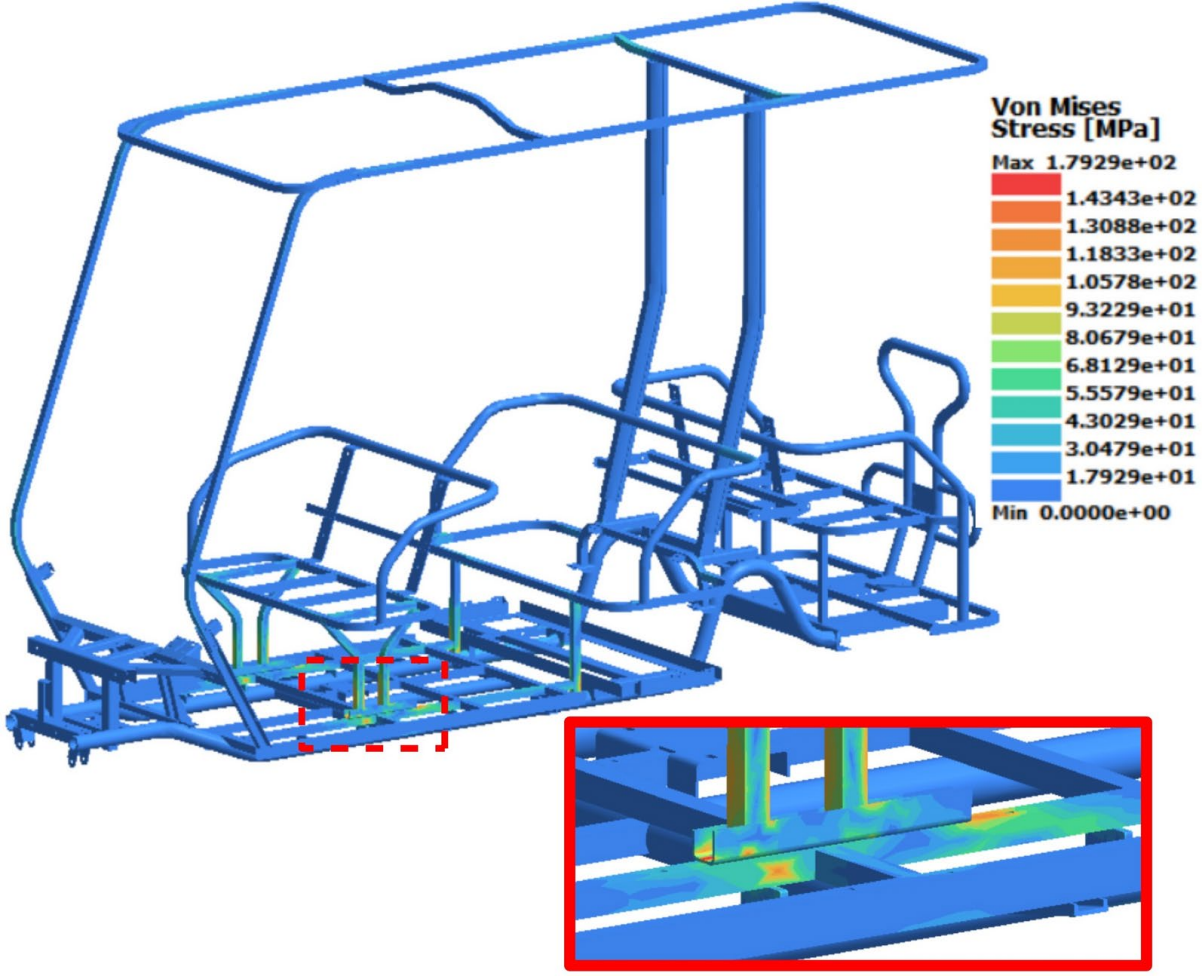
Stress distribution results of the torsional loading study.

**Fig. 18:**
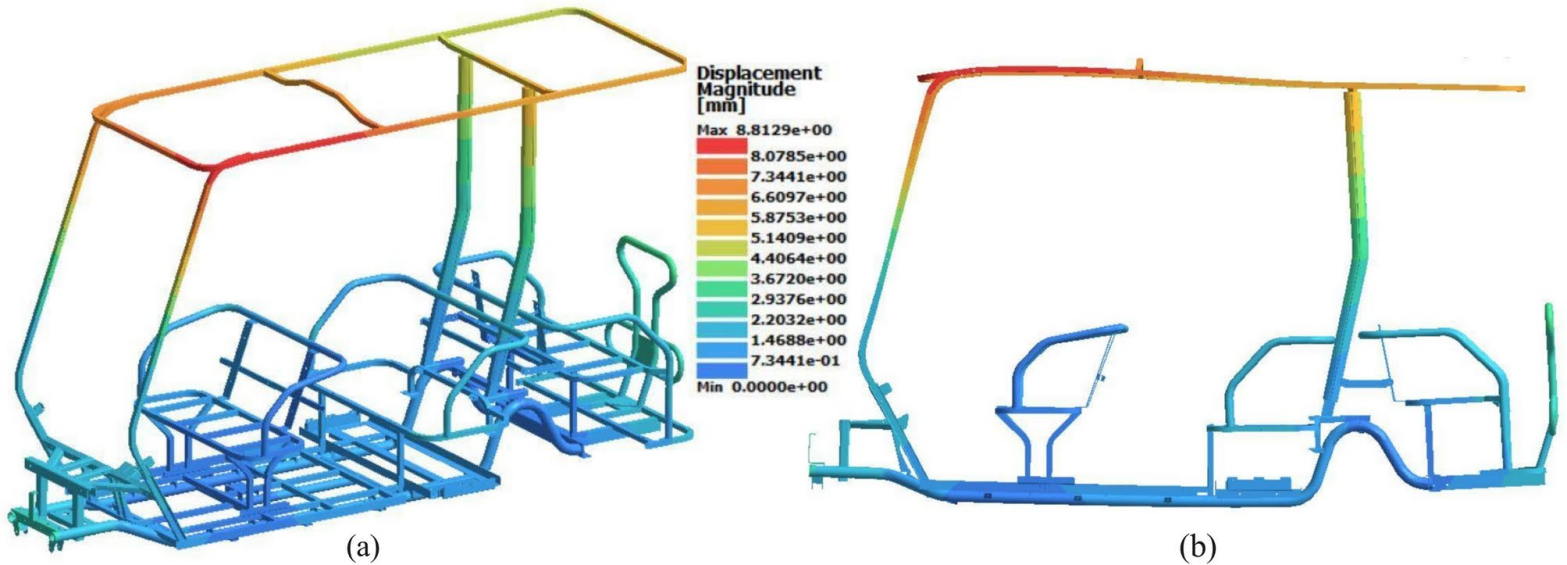
Displacement results of the torsional loading study. (**a**) Isometric view (deformation scale 1:1), (**b**) Side view (deformation scale 20:1).

The effect of torsional loading was significant due to the asymmetry in stress distribution and displacement results between the right and left parts of the chassis. The maximum von Mises stress reached 179 MPa at 0.015 s, as illustrated in Fig. 17, with stress concentrations observed on the left side of the front seat base. This stress value is below the yield strength of the material used, resulting in a safety factor of 2.07. The maximum displacement recorded was 8.8 mm at 0.24 s, predominantly located on the front left part of the roof, as depicted in Fig. 18. Table 9 summarizes the results of the dynamic analysis.

**Table 9:** Summary of the dynamic analysis results.

Study	Maximum stress (MPa)	Maximum displacement (mm)	Safety factor
Front Loading	288	15.9	1.28
Rear Loading	174	5.1	2.12
Torsional Loading	179	8.9	2.07

The safety factors calculated indicate that the chassis undergoes only elastic deformation during the dynamic analysis, confirming the structural safety of the chassis. However, these stress results highlight the need to reinforce specific parts of the chassis, including the front seat base and rear shield, to further enhance the performance of the chassis under dynamic loading conditions, and increase the dynamic safety factor.

### Static analysis

**Fig. 19:**
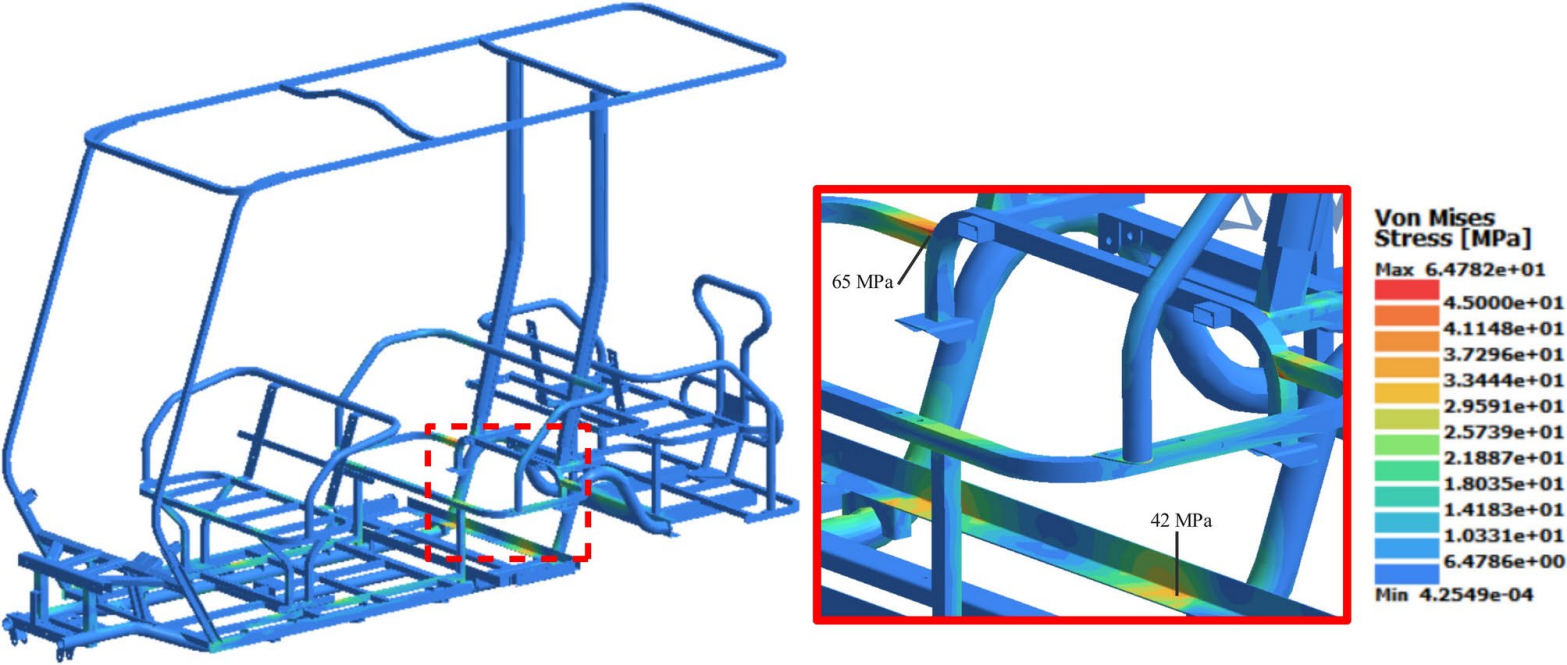
Stress distribution results of static analysis.

**Fig. 20:**
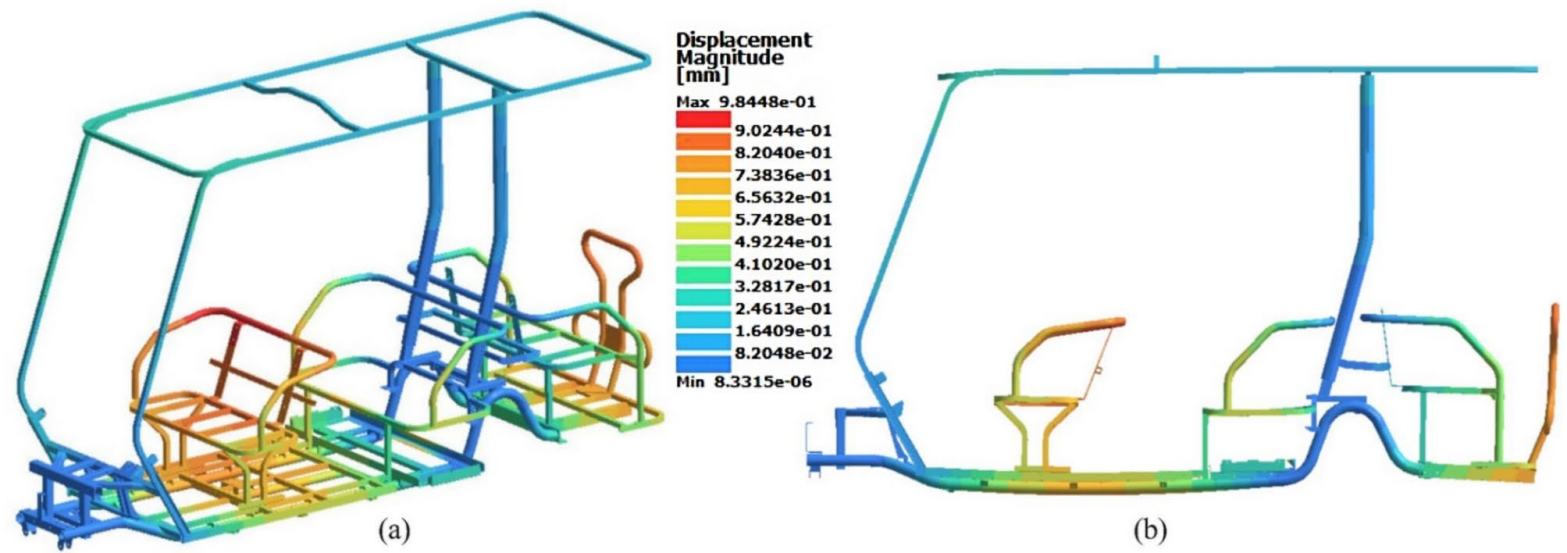
Displacement results of static analysis. (**a**) Isometric view (deformation scale 1:1), (**b**) Side view (deformation scale 60:1).

The von Mises stress results show the existence of stress concentration areas on the square tubes of the back seat, with a maximum value of approximately 65 MPa, as shown in Fig. 19. However, this value is much lower than the yield strength of the square tube material and results in a static safety factor of 5.69. Figure 20 shows that the maximum displacement was nearly 1 mm, which reflects the high bending stiffness and rigidity of the chassis. It can be observed that vertical downward displacement is concentrated at the middle and end of the chassis. This is due to the boundary conditions applied, assuming that the chassis is simply supported at the shock towers.

Table 10 summarizes the values of the safety factors obtained from static and dynamic studies. By comparing the static safety factor with the highest dynamic safety factor, a load factor of 4.44 between dynamic and static loads is calculated. This factor quantifies the increased stress that the chassis experiences under dynamic loads compared to that under static loads. This factor is vital for design considerations, indicating that the chassis must withstand forces 4.44 times greater than static loads during dynamic events such as hitting speed bumps.

**Table 10:** Safety factor results.

Analysis		Von-misses stress (MPa)	Safety factor
Dynamic	Front loading	288	1.28
	Rear loading	174	2.13
	Torsional loading	179	2.07
Static	65	5.69	

The calculated dynamic load factor of 4.44 closely aligns with the recommended dynamic load factors for similar loading scenarios, as outlined in^1^. This reference emphasizes the importance of modeling and analyzing transient loads in worst-case scenarios, which generate dynamic loads significantly greater than static loads. Consequently, designers often use load factors based on static wheel loads. For dynamic loading events, such as when a vehicle encounters a front or rear bump, a vertical acceleration of 4g is recommended at the affected wheel, where g represents gravitational acceleration. The resulting forces on the wheel assembly (unsprung mass) and chassis (sprung mass) are then calculated to evaluate the amplified stresses on the chassis. The slight difference between the calculated load factor and the recommended value arises from the specific vehicle parameters and modeling assumptions used in this study. This comparison validates the methodology employed while acknowledging the influence of scenario-specific dynamics on the calculated load factor.

Additionally, our findings are further supported by the safety factor (design factor) recommendations for ductile materials, as discussed in^52^. The recommended safety factor depends on the level of uncertainty in the design data and loading conditions. For static structures under dynamic loading with significant uncertainty about loads and stress analysis, a safety factor of 4.0 or higher is advised.

Furthermore, in comparison with the findings reported by Kishan et al.^22^, a dynamic load factor of approximately 5 can be deduced from the stress results. The similarity between the dynamic load factors demonstrates consistency between the two studies, despite differences in vehicle type, chassis geometry, and analysis methodologies. This consistency reinforces the validity of our findings and underscores the importance of accounting for dynamic loads in chassis design, and explains how dynamic loads significantly amplify the stresses on a chassis compared to static loads. Our calculated load factor of 4.44 provides a refined and realistic estimate derived from real-world variables, including bump dimensions and vehicle speeds.

These benchmarks, derived from both theoretical recommendations and empirical studies, provide strong support for the achieved dynamic safety factor in this study and ensure that the chassis can withstand amplified stresses with a significant margin of reliability, meeting the safety standards required for electric vehicles.

## Conclusion

In this paper, static and dynamic analyses were conducted on an electric vehicle chassis using finite element analysis with SimSolid software. A quarter vehicle model was adopted to study the dynamic suspension force transmitted to the chassis when the vehicle hit a speed bump. The mathematical model was simulated using MATLAB Simulink software, and different suspension force values were evaluated by varying the vehicle speed with standard bump dimensions. Three dynamic studies were conducted: Front Loading, Rear Loading, and Torsional Loading. The maximum suspension force calculated at each wheel was 3170 N, resulting in a maximum von Mises stress of 288 MPa and a safety factor of 1.28 in the front loading study. In the static study, the maximum von Mises stress was 65 MPa with a safety factor of 5.69. This significant difference in safety factors between static and dynamic studies underscores the importance of considering dynamic loads in chassis design. Our findings suggest a dynamic load factor of 4.44, which can serve as a benchmark for assessing the structural performance and durability of electric vehicle chassis under similar dynamic loading conditions.

## Supplementary Information

Supplementary Information 1.

## Data Availability

The datasets used and/or analysed during the current study available from the corresponding author on reasonable request.
