# Supplementary figures and images for: Structural performance evaluation of electric vehicle chassis under static and dynamic loads
Source: Sci Rep. 2025 Feb 12;15:5168. doi: 10.1038/s41598-025-86924-w (PMC11821887; doi:10.1038/s41598-025-86924-w)

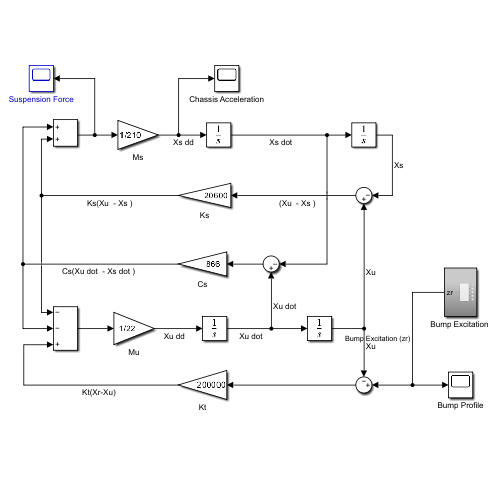

Supplement: Supplementary file 1 — Supplementary Information 1. [file 41598_2025_86924_MOESM1_ESM.slx › metadata/thumbnail.png]
